# Pulmonary amyloidosis with calcified nodules and masses - a six-year computed tomography follow-up: a case report

**DOI:** 10.4076/1757-1626-2-6540

**Published:** 2009-09-08

**Authors:** Isabela Garcia Vieira, Edson Marchiori, Gláucia Zanetti, Rafael Ferracini Cabral, Tatiana Chinem Takayassu, Gabriela Spilberg, Raquel Ribeiro Batista

**Affiliations:** 1 Service of Diagnostic Radiology, Clementino Fraga Filho Universitary HospitalRua Prof. Rodolpho Paulo Rocco, 225. CEP 21.941.913. Cidade Universitária. Rio de JaneiroBrazil; 2 Department of Radiology, Faculty of Medicine, Fluminense Federal UniversityRua Marquês do Paraná, 303. CEP 24.033.900. Niterói. Rio de JaneiroBrazil

## Abstract

**Introduction:**

Pulmonary amyloidosis is an uncommon disease, characterized by extracellular deposition of fibrillary protein in the lungs. It appears in three forms: tracheobronchial, nodular pulmonary, and alveolar septal. There are few reports of long-term observation of primary pulmonary amyloidosis.

**Case presentation:**

We present the case of a 47-year-old man who presented with fever, dyspnea, cough and hemoptysis. Chest radiograph and computed tomography revealed multiple pulmonary nodules and masses. The patient underwent open lung biopsy, which diagnosed pulmonary amyloidosis.

**Conclusion:**

Pulmonary nodular amyloidosis should be considered in the differential diagnosis of pulmonary nodules or masses.

## Introduction

Amyloidosis is a systemic disease caused by extracellular accumulation of amyloid [1]. It can be idiopathic (primary form) or associated with various inflammatory, hereditary, or neoplastic pathogeneses (secondary or reactive form). Pulmonary amyloidosis may be part of a widespread process that involves many organs, or it may be localized to the airways and lung parenchyma.

Primary pulmonary amyloidosis is a localized form of amyloidosis that is confined to the lung parenchyma [2-5]. Unlike systemic amyloidosis, localized pulmonary amyloidosis usually follows a benign course [6]. It can occur in three forms: diffuse interstitial deposits, single or multiple pulmonary nodules, or, most commonly, submucosal tracheobronchial deposits [3-5,7,8]. Although pulmonary nodular amyloidosis is a rare disease [2], it should be considered in the differential diagnosis of pulmonary nodules or masses, mainly with primary or metastatic neoplasms and granulomatous diseases [2], and particularly with pulmonary hyalinizing granulomas [4,7,9].

## Case presentation

A 47-year-old Caucasian man presented with a three-week history of progressive dyspnea, productive cough, and weight loss of 10 kg in the preceding 3 months. He suffered from several episodes of respiratory infection in preceding months that improved after antibiotic therapy. He smoked 16 packs per year.

Upon admission, physical examination showed an ill looking patient who was thin, pale, acyanotic and tachycardic. His blood pressure was 100/70 mmHg, his pulse 100 beats/min, and his respiratory rate was 26 breaths/min. There was no cervical, axillary, or inguinal lymphadenopathy. Lung auscultation revealed diffusely scattered crackles and rhonchi bilaterally. A physical examination of the heart and abdomen revealed no abnormal findings.

Laboratory tests showed the following blood analysis results: red blood cell count, 4.7 milion/mm^3^; hemoglobin, 11.1 g/dL; hematocrit, 34.8%; white blood cells, 4,300/mm^3^; and platelets, 200,000/mm^3^. Arterial blood gas analysis while breathing room air showed an arterial oxygen tension (PaO_2_) of 80 mmHg and an arterial carbon dioxide tension (PaCO_2_) of 32 mmHg. Blood analysis further revealed the following molecular concentrations: sodium, 136 mEq/L; potassium, 4.5 mEq/L; blood urea nitrogen, 26 mg/dL; and creatinine, 1.1 mg/dl. Liver function tests were normal. Serology for HIV and tests for acid-fast bacilli and fungi in three sputum samples were negative. Respiratory function tests demonstrated a moderate obstructive disease. Echocardiography revealed a normal ejection fraction with no myocardial or valvular abnormalities.

**Figure 1.**
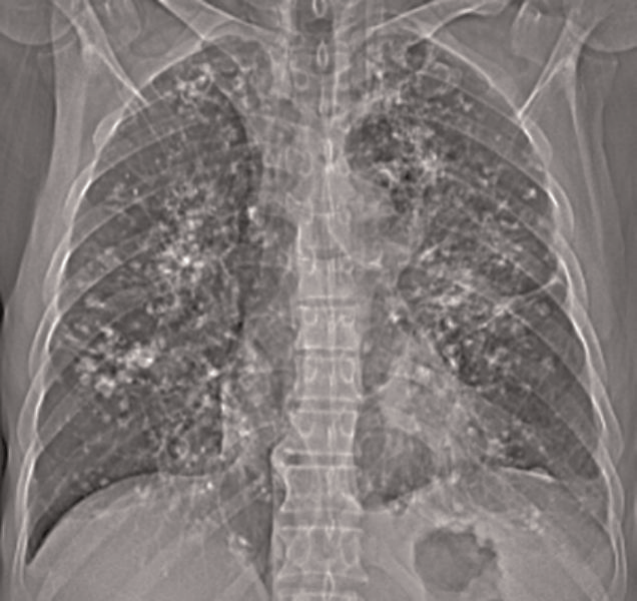
Chest radiograph demonstrating diffuse pulmonary opacities with extensive foci of calcification.

**Figure 2.**
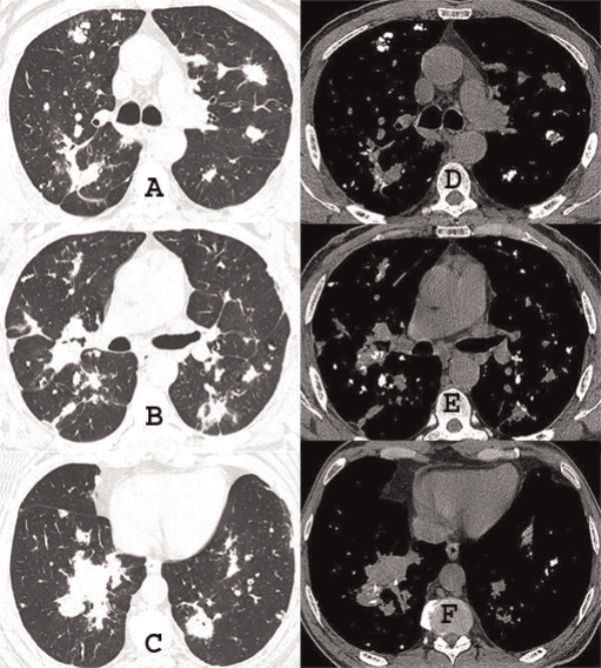
Chest CT obtained with lung window settings **(A, B and C)**, revealing nodules and masses, and irregular contours in both lungs. CT images obtained with mediastinal window settings **(C, D and E)** show pulmonary lesions permeated by calcifications.

**Figure 3.**
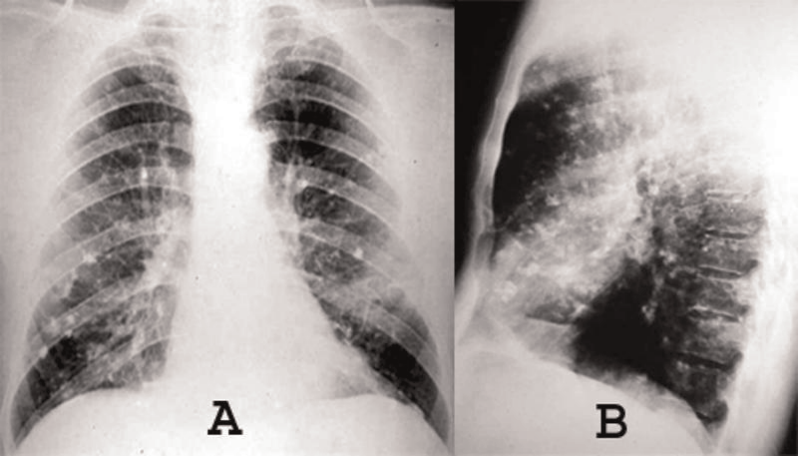
Posteroanterior **(A)** and lateral chest radiograph **(B)**, performed six years earlier already showed the presence of calcified nodules, which were smaller than in recent radiographs.

**Figure 4.**
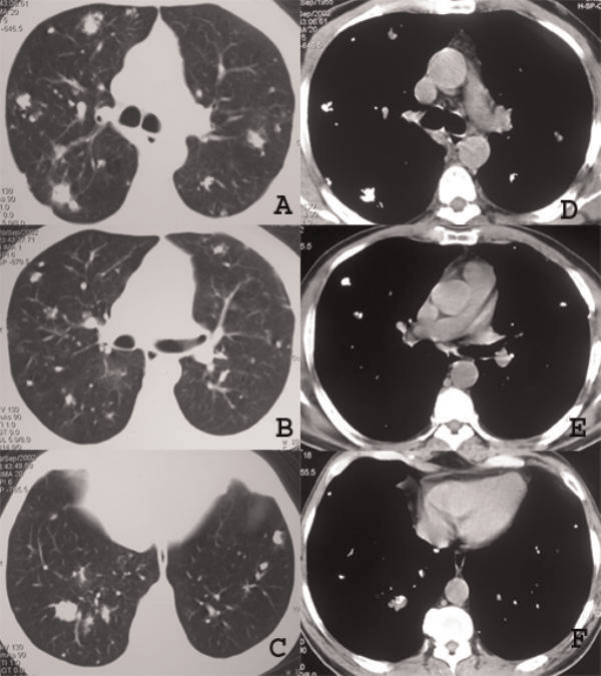
CT of the thorax performed in the same period as the radiographs in Figure 3, demonstrating scattered pulmonary nodules, including several with calcifications.

A chest radiograph (Figure 1) and computed tomography (CT) on the thorax (Figure 2) showed multiple pulmonary nodular opacities and masses of soft tissue density that had irregular contours, and were diffusely and bilaterally distributed. Most of the nodules and masses contained calcifications, but none were cavitated. Radiographs (Figure 3) and CT of the thorax (Figure 4) had been performed at another institution six years prior, showing nodular lesions with calcifications that were smaller than at present. Since he was asymptomatic at the time, he did not return for further examinations.

Bronchoscopy showed no abnormalities. A bronchoalveolar lavage was negative for tuberculosis, fungi or malignancy. An open lung biopsy was performed and histopathological analysis showed pulmonary amyloidosis. Three months later, the patient died from a respiratory infection.

## Discussion

Diagnosis of amyloidosis is difficult owing to its diverse clinical presentation [9]. Amyloid proteins can infiltrate virtually all organ systems. Pulmonary involvement is most commonly observed in the localized (primary) form of amyloidosis [4,7,10]. In this form, males are more affected than females. The mean age of onset is 55-60 years and common sites of involvement include the kidneys, lungs, gastrointestinal tract, and skin [7]. The respiratory system is involved in approximately 50% of patients diagnosed with amyloidosis, although radiographic demonstration is much less common [7,8]. As described in detail below, three patterns of involvement have been described: tracheobronchial, diffuse parenchymal, and nodular [3-5,7,8].

Tracheobronchial amyloidosis generally presents with symptoms of airway obstruction, such as dyspnea or cough, hemoptysis, and recurrent pneumonia [9,11]. Classic radiological signs of tracheobronchial disease include nodular and irregular narrowing of the tracheal lumen, airway wall thickening, and calcified amyloid deposits. Lobar or segmental collapse may be seen with endobronchial obstruction from amyloid deposition [4,5,7,9].

Diffuse parenchymal pulmonary amyloidosis is the rarest form of respiratory amyloidosis [7,9,14]. It usually presents with dyspnea, cough, hemoptysis, or a combination of these symptoms. The deposition of amyloid protein causes alveolar membrane and capillary damage, which ultimately leads to impaired gas exchange. Radiologically, the diffuse parenchymal and alveolar septal forms of amyloid deposits appear as nonspecific diffuse interstitial or alveolar opacities, and, once established, remain stable over time. The abnormal areas can calcify [14]. A high-resolution CT scan of the thorax can reveal interlobular septal thickening or irregular lines that frequently have a predominant basilar and peripheral distribution [13], as well as small, well-defined nodules (2-4 mm in diameter), and confluent consolidations located predominantly in the subpleural regions. Some nodules may show calcifications, and some of the areas of consolidation may contain punctate foci of calcification [2]. When these interstitial findings predominate, the differential diagnosis includes idiopathic pulmonary fibrosis, scleroderma, and rheumatoid lung disease [7,13,14].

Nodular parenchymal amyloid deposits usually appear in multiple sites; focal deposits do occur, albeit much less commonly. The patients are generally asymptomatic. Amyloid nodules are generally localized to the lower lobes, in the peripheral and subpleural areas [12]. They have four characteristic features on CT: (i) sharp, lobulated contours; (ii) calcification, often central or in an irregular pattern within the nodule (seen in about 50% of cases) [2,4,7,8,12]; (iii) multiple shapes and sizes varying from 0.5 to 15 cm [2,4]; and (iv) slow growth, often over years, with no regression [4,7,12]. Cavitation is very rare [13].

CT is especially helpful in the demonstration of subtle calcification, which is often the only finding that can suggest the diagnosis. Calcification is seen in approximately half the cases [4,7,12]. The differential diagnosis includes primary or metastatic neoplasm and granulomatous diseases [2,4,7,9]. Tissue biopsy is essential for a definitive diagnosis [9]. Adenopathy occurs predominantly in systemic amyloidosis. This finding may be isolated or more commonly, associated with parenchymal involvement [13]. The lymph nodes may have calcifications [10,14].

The diagnosis of amyloidosis usually requires histological confirmation. Histopathological diagnosis is made by the finding of amyloid, which is an inert, proteinaceous, homogeneous, acellular, and eosinophilic material that, when subjected to histochemical staining with Congo red shows green birefringence under polarized light [2,4,11-13]. This immunohistochemistry method remains the gold standard of diagnosis [12].

Management of localized pulmonary amyloidosis is dependent on the severity of symptoms. Asymptomatic patients may not require treatment [9,12]. Because of the relative rarity of localized pulmonary amyloidosis, there is a lack of randomized controlled trials. Various reported modalities of treatment include bronchoscopic resection, surgical resection, carbon dioxide laser ablation, and Nd:YAG laser therapy [12,15]. Parenchymal amyloid nodules generally grow slowly and remain asymptomatic. In such cases, treatment is generally not needed. Surgical resection may be considered; however, there is a possibility of relapse [9].

## Abbreviations

CT: computed tomography
PaCO2: arterial carbon dioxide tension
PaO2: arterial oxygen tension

## References

[bib-001] Esteban MB, Obrer AA, Martínez AH, Guerrero ME, Armengod AC (2007). Pulmonary nodular amyloidosis. Cir Esp.

[bib-002] Kim HY, Im JG, Song KS, Lee KS, Kim SJ, Kim JS, Lee JS, Lim TH (1999). Localized amyloidosis of the respiratory system. J Comput Assist Tomogr.

[bib-003] Slanetz PJ, Whitman GJ, Shepard JA, Chew FS (1994). Nodular pulmonary amyloidosis. AJR Am J Roentgenol.

[bib-004] Marchiori E, Souza AS, Ferreira A, Azevedo KC, Fialho SM, Crespo SJV (2003). Amiloidose pulmonar: aspectos na tomografia computadorizada. Radiol Bras.

[bib-005] Prince JS, Duhamel DR, Levin DL, Harrell JH, Friedman PJ (2002). Nonneoplastic lesions of the tracheobronchial wall: radiologic findings with bronchoscopic correlation. RadioGraphics.

[bib-006] Suzuki H, Matsui K, Hirashima T, Kobayashi M, Sasada S, Okamato N, Kitai N, Kawahara K, Fukuda H, Komiya T, Kawase I (2006). Three cases of the nodular pulmonary amyloidosis with a long-term observation. Intern Med.

[bib-007] Urban BA, Fishman EK, Goldman SM, Scott WW, Jones B, Humphrey RL, Hruban RH (1993). CT evaluation of amyloidosis: spectrum of disease. Radiographics.

[bib-008] Brown K, Mund DF, Aberle DR, Batra P, Young DA (1994). Intrathoracic calcifications: radiographic features and differential diagnoses. Radiographics.

[bib-009] Gaurav K, Panda M (2007). An uncommon cause of bilateral pulmonary nodules in a long-term smoker. J Gen Intern Med.

[bib-010] Utz JP, Swensen SJ, Gertz MA (1996). Pulmonary amyloidosis: the Mayo Clinic experience from 1980-1993. Ann Intern Med.

[bib-011] dos Santos JW, Schneider Filho A, Bertolazzi A, Michel GT, da Silva LV, Melo CR, Pedro VD, Spilmann D, Figaro JK (2008). Primary tracheobronchial amyloidosis. J Bras Pneumol.

[bib-012] Gillmore JD, Hawkins PN (1999). Amyloidosis and the respiratory tract. Thorax.

[bib-013] Pickford HA, Swensen SJ, Utz JP (1997). Thoracic Cross-Sectional Imaging of Amyloidosis. AJR Am J Roentgenol.

[bib-014] Graham CM, Stern EJ, Finkbeiner WE, Webb WR (1992). High-Resolution CT appearance of Diffuse Alveolar Septal Amyloidosis. AJR Am J Roentgenol.

[bib-015] Nugent AM, Elliott H, McGuigan JA, Varghese G (1996). Pulmonary amyloidosis: treatment with laser therapy and systemic steroids. Respir Med.

